# Environmental and health-related research on application and production of rare earth elements under scrutiny

**DOI:** 10.1186/s12992-022-00879-5

**Published:** 2022-10-17

**Authors:** Doris Klingelhöfer, Markus Braun, Janis Dröge, Axel Fischer, Dörthe Brüggmann, David A. Groneberg

**Affiliations:** 1 Institute of Occupational, Social and Environmental Medicine, Goethe University, Theodor-Stern-Kai 7, 60590 Frankfurt, Germany; 2 Clinical Research Unit of Allergy, Institute of Occupational Medicine, Charité University Berlin, Berlin, Germany

**Keywords:** REE, Risks, Market drivers, Research output, Gadolinium, Cerium, Neodymium

## Abstract

**Background:**

Unlike most other commodities, rare earth elements (REEs) are part of a wide range of applications needed for daily life all over the world. These applications range from cell phones to electric vehicles to wind turbines. They are often declared as part of “green technology” and, therefore, often called “green elements”. However, their production and use are not only useful but also risky to the environment and human health, as many studies have shown. Consequently, the range of global research efforts is broad and highly variable, and therefore difficult to capture and assess. Hence, this study aims to assess the global parameters of global research on REE in the context of environment and health (REE_eh_). In addition to established bibliometric parameters, advanced analyses using market driver and scientific infrastructure values were carried out to provide deep insight into incentives, necessities, and barriers to international research.

**Results:**

The focus of REE research is in line with national aspirations, especially from the major global players, China and the USA. Whereas globally, regional research interests are related to market interests, as evidenced by the inclusion of drivers such as electric vehicles, wind turbines, and permanent magnets. The topics receiving the most attention are related to gadolinium used for magnetic resonance imaging and the use of ceria nanoparticles. Since both are used for medical purposes, the medical research areas are equally profiled and mainly addressed in high-income countries. Nevertheless, environmental issues are increasingly in focus.

**Conclusions:**

There is still a need for research that is independent and open-ended. For this, market-independent technologies, substitutes and recycling of REEs need to be addressed scientifically. The results of this study are relevant for all stakeholders, from individual scientists to planners to funders, to improve future research strategies in line with these research mandates.

**Graphical Abstract:**

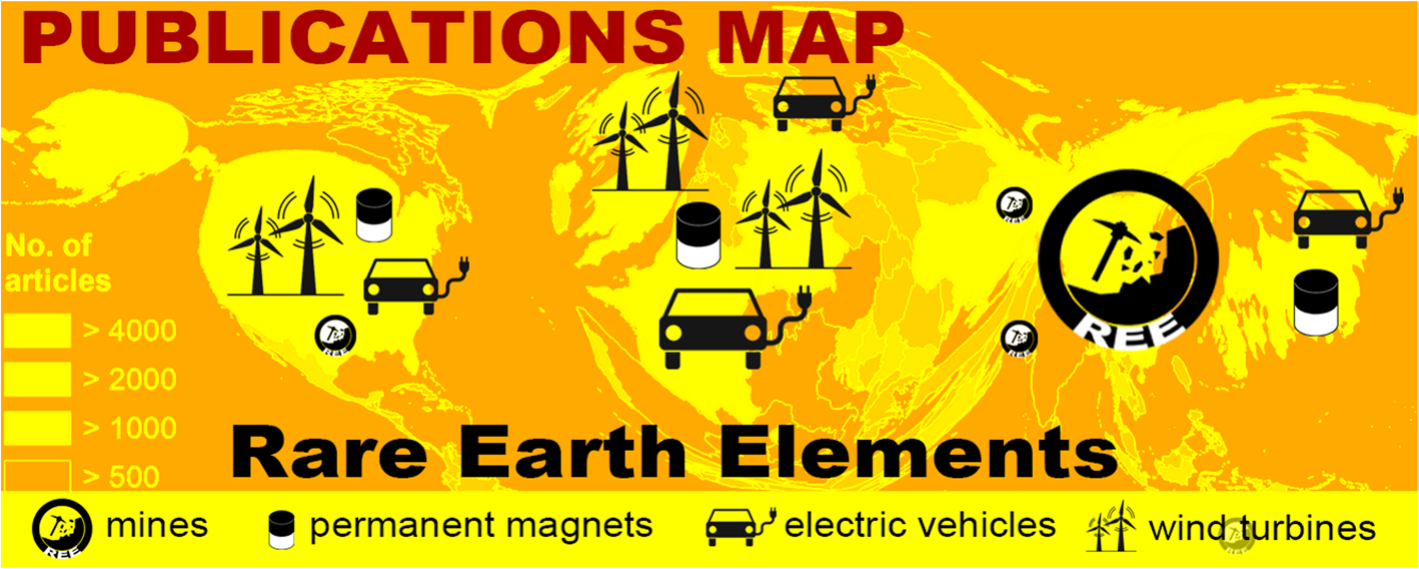

## Background

Entire daily lives are impacted by rare earth elements (REEs) as they are found in every car, computer, cell phone, television, and energy-efficient fluorescent light bulb, naming just the most common applications [1]. This exacerbates the often overlooked fact that they are emerging contaminants (ECs), as they are currently unregulated for humans and the environment, not included in environmental and public health programs, are micropollutants with very low detection limits. Their mechanisms of toxicity are poorly understood [2].

According to the *International Union of Pure and Applied Chemistry* (IUPAC), they form a homogenous group of 17 chemical elements forms the REEs, which show a coherent behavior [1, 3]. In addition to the 15 lanthanides, scandium and yttrium also belong to this group. Although a distinction is generally made between light and heavy REEs, there is no consistent definition worldwide. The heavy REEs occur less frequently, have higher atomic masses and are characterized by lower solubility and alkalinity [4]. REEs are not as rare as their designation suggests. Instead, they occur in significant abundance in almost all rock formations [4]. Therefore, the designation as rare elements is actually misleading. It is not based on their geological rarity in the earth’s crust but on their occurrence in the mineral mixture in low concentrations as well as the few occurrences that can be mined economically [5]. However, when converted to atomic concentrations, especially the REEs with odd atomic numbers are rarer, including those of great value [1].

REEs are highly reactive because they are very easily oxidized. Therefore, they are used as reducing agents for difficult to reduce metals [2]. The exceptional applicability for technical and industrial usages is due to their specific mechanical, chemical, optical, and magnetic characteristics [5]. They are widely used in high technology but also in traditional applications. They are key components for many high-tech applications, reaching from everyday applications to power generation, chemistry, mobility, medicine, defense, and agriculture [5]. Practical applications also provide environmental benefits, such as phosphate removal from aquatic systems to prevent eutrophication with lanthanum hydroxide [6] or lanthanum carbonate groups [7] or photocatalytic applications such as pollutant degradation, carbon dioxide reduction, and hydrogen evolution [8]. Given all these uses, people are often exposed to them [2].

The difficult separation of the elements remains a challenge to this day. Additionally, the eminently increasing use in technical devices raises concerns about the sustainability of REEs, which has stimulated numerous studies on the subject [5]. This difficulty is the cause of the high price of REEs, which is disproportionate to their deposits. In addition, high demand and tight supply due to limited mining areas in China contribute to their value. Sufficient global supply of REEs as critical metals is thus on everyone’s lips [1]. Moreover, recycling rates are still exceedingly low, so that a desirable circular economy unfeasible is currently not feasible [5].

The increasing use of REEs for industrial applications implicates a corresponding increase in health and environmental impacts. As anthropogenic compounds, they enter soils and waters through disposal, discharges from mining operations, wastewater, and atmospheric emissions. Further dispersal in the environment occurs through wind blowing, runoff, leaching, or irrigation, releasing, for example, radioactivity or other chemical byproducts [2, 9]. Its use as a component of fertilizers and animal feeds also contributes to its widespread use. It is based on the dose-response effect. At low doses it has a growth-prompting effect, while at high doses it is toxic [10, 11].

Adverse health effects on aquatic and terrestrial organisms have been already reported [12–14]. However, there is a need for more comprehensive toxicological studies, as the transfer of effects to species or to ecosystems is still not well understood [2]. Another application of REEs is in medicine, e.g., gadolinium as a contrast agent for magnetic resonance imaging (MRI) and yttrium for radioimmunotherapy of non-Hodgkin’s lymphoma (NHL). However, exposure to these REEs has also been shown to be risky. For example, the use of gadolinium in contrast agents has been associated with nephrogenic systemic fibrosis [15], and its accumulation in the brain can cause adverse effects on the central nervous system [16]. Other reported health effects associated with REE exposure include the occurrences of pneumoconiosis, anti-testicular effects, male sterility, dysfunctional neurological disorders, genotoxicity, and fibrotic tissue damage. However, studies on human health effects are limited, particularly concerning the toxicity of the individual REEs and the long-term effects of their exposure [2]. In addition, knowledge of environmental occurrence and behavior is low, which is especially on demand for developing countries with their potential REE sources [17], as data is mostly generated in developed countries [2].

Therefore, an analysis of the scientific landscape of REEs related to environmental and human health (REE_eh_), as intended in the present study, is useful and important. Scientists can benefit from it to identify, plan, and expand current and future projects. Policymakers and funders can benefit by being able to support scientific efforts more specifically. Thus, the goal is to present the scientific basis on REEs in terms of their impact on the environment and human health by assessing general and advanced features of the research output. That will provide information on the drivers and barriers to the research conducted, as well as the incentives and requirements for future research, and identify and assess key player of global research on REE_eh_.

## Results

For the overall publication output on REE_eh_, 6941 articles (n) could be retrieved from WoS.

The majority of articles were written in English (98.44%, *n* = 6833). Only a few articles were published in other languages, e.g., Chinese (*n* = 32), German (*n* = 17), and Spanish (*n* = 16), to name those that contributed more than 15 articles.

### Research areas

**Fig. 1:**
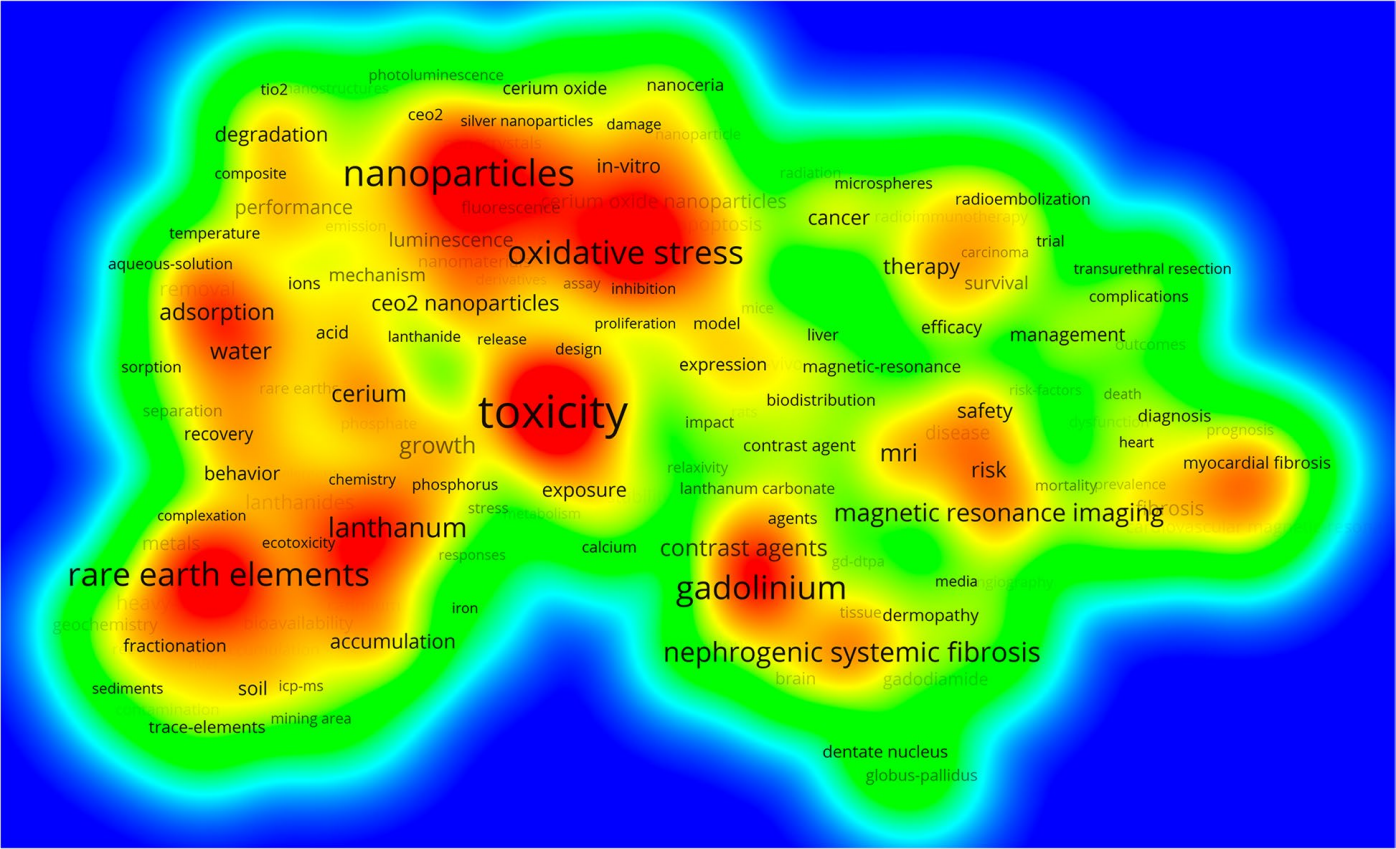
Density cluster analysis of occurring keywords (threshold: 50 occurrences)

In addition to the title synonymous term *rare earth elements*, the densest keyword clusters were led by the terms *toxicity*, *gadolinium*, *lanthanum*, *nanoparticles*, and *adsorption*. Lower density clusters were identified for research areas around the keywords *therapy and safety* and *late gadolinium enhancement and fibrosis*. These clusters identify the main areas of REE_eh_ research, which include the effect of cerium oxide nanoparticles (CeNPs), the adverse effects of gadolinium as a contrast agent in magnetic resonance imaging, and accumulation processes in soil, plants, and water (Fig. 1).

In accordance with these results, the five most frequently assigned WoS categories relating to the different research areas were *Chemistry*, *Materials Science, Environmental Sciences & Ecology*, *Radiology Nuclear Medicine & Medical Imaging*, and *Engineering* (Table 1).

**Table 1:** Most assigned WoS categories measured by the number of articles

WoS category	Articles	Citations	Citation rate
Chemistry	1165	24,644	21.15
Materials Science	928	26,357	28.40
Environmental Sciences & Ecology	901	20,824	23.11
Radiology, Nuclear Medicine & Medical Imaging	852	31,123	36.53
Engineering	573	16,653	29.06
Science & Technology – Other Topics	449	13,261	29.53
Cardiovascular System & Cardiology	445	15,736	35.36
Physics	389	9141	23.50
Toxicology	336	8711	25.93
Urology & Nephrology	304	8053	26.49

### Analysis of publication development over time

**Fig. 2:**
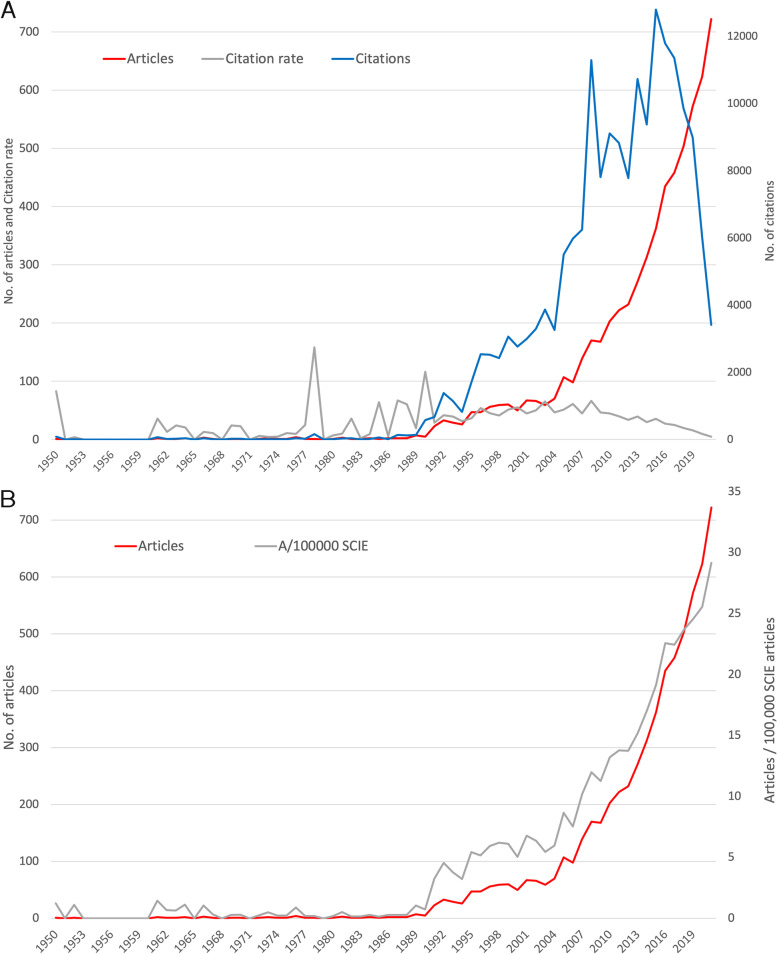
Publication development over time. **A** Number of REE_eh_ articles, number of citations, and citation rate. **B** Number of REE_eh_ articles and articles per 100,000 SCIE (Science Citation Index Expanded) articles. The horizontal gray curve shows that the proportion of REE_eh_ articles to all SCIE articles is nearly constant until 1990, while the steep increase after 1990 shows that the proportion of REE_eh_ articles increases much more than the number of SCIE articles

The first article found about REE_eh_ was published in 1950. In the following years, there were no or only a few publications. It was only since the 1990s that the annual number of articles increased to double digits and since 2005 the number increased to triple digits with a steep increase since then. The previous peak was reached in 2021 with *n* = 722 articles on REE_eh_. The number of annual citations (c) increased similarly since the 1990s, reaching two major peaks in 2008 (c = 11,288) and 2015 (c = 12,798). Afterward, a steep decline could be observed. The average annual citation rate (cr) emphasizes the year with high citation numbers and small publication volumes, leading to the peaks of 1978, 1987, and 1990 (Fig. 2A). A comparison of the increase in publication output of REE_eh_ and all SCIE (*Science Citation Index Expanded*) articles, measured by the ratio *Articles / (SCIE articles / 100,000)*, shows the above-average growth of articles on REE_eh_ (Fig. 2B).

**Fig. 3:**
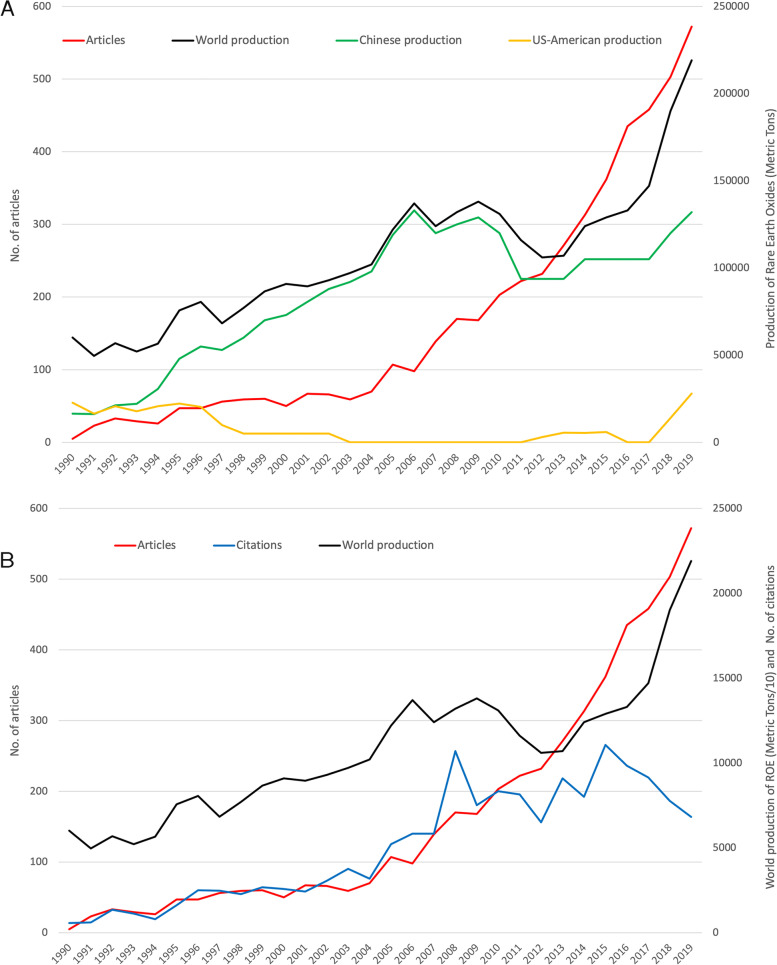
Comparison of development of articles on REE_eh_ and world production of rare earth oxides equivalents (Metric Tons). **A** Number of articles, world production, Chinese production, and US-American production. **B** Number of articles, number of citations, and world production [18]

The comparison of the development of the publication figures and the production of rare earth oxides over time since 1990 [18] show a steady increase for both (Fig. 3A). Chinese mine production equals global production and accounts for by far the largest share. Until 2002, when mining ceased for the time being, the USA accounted for a visible share of production. The period of short-term decline in mining starting in 2009, leading to the low point in 2012, was also accompanied by a decline in citation numbers over the same period, reaching the same low point in 2012. Production then picked up again, also in the USA, mining resumed, and publication patterns also grew disproportionately again (Fig. 3B).

Table 2 lists the most frequently cited articles. The article that has received the most citations so far is on the toxicity of cerium oxide nanoparticles, among others, by Xia et al. and was published in 2008 [19].

**Table 2:** Ten most cited articles on REE_eh_

Authors	Country	Year	Citations	Title	Journal
Xia, T. et al.	USA, Germany	2008	1807	Comparison of the Mechanism of Toxicity of Zinc Oxide and Cerium Oxide Nanoparticles Based on Dissolution and Oxidative Stress Properties	ACS nano
Grobner, T.	Austria	2006	1284	Gadolinium - a specific trigger for the development of nephrogenic fibrosing dermopathy and nephrogenic systemic fibrosis?	Nephrology, Dialysis, Transplantation
McDonald, R.J. et al.	USA	2015	891	Intracranial Gadolinium Deposition after Contrast-enhanced MR Imaging	Radiology
Witzig, T.E. et al.	USA	2002	846	Randomized controlled trial of yttrium-90-labeled ibritumomab tiuxetan radioimmunotherapy versus rituximab immunotherapy for patients with relapsed or refractory low-grade, follicular, or transformed B-cell non-Hodgkin’s lymphoma	Journal of Clinical Oncology
McCrohon, A. et al.	UK, Germany	2003	754	Differentiation of heart failure related to dilated cardiomyopathy and coronary artery disease using gadolinium-enhanced cardiovascular magnetic resonance	Circulation
Salem, R. et al.	USA	2010	684	Radioembolization for Hepatocellular Carcinoma Using Yttrium-90 Microspheres: A Comprehensive Report of Long-term Outcomes	Gastroenterology
Celardo, I. et al.	Italy, Japan	2011	656	Pharmacological potential of cerium oxide nanoparticles	Nanoscale
Heckert, E.G. Et al.	USA	2008	655	The role of cerium redox state in the SOD mimetic activity of nanoceria	Biomaterials
Kanda, T. et al.	Japan	2015	586	Gadolinium-based Contrast Agent Accumulates in the Brain Even in Subjects without Severe Renal Dysfunction: Evaluation of Autopsy Brain Specimens with Inductively Coupled Plasma Mass Spectroscopy	Radiology
Schubert, D. et al.	USA	2006	551	Cerium and yttrium oxide nanoparticles are neuroprotective	Biochemical and Biophysical Research Communications

### Analysis of publication countries

**Fig. 4:**
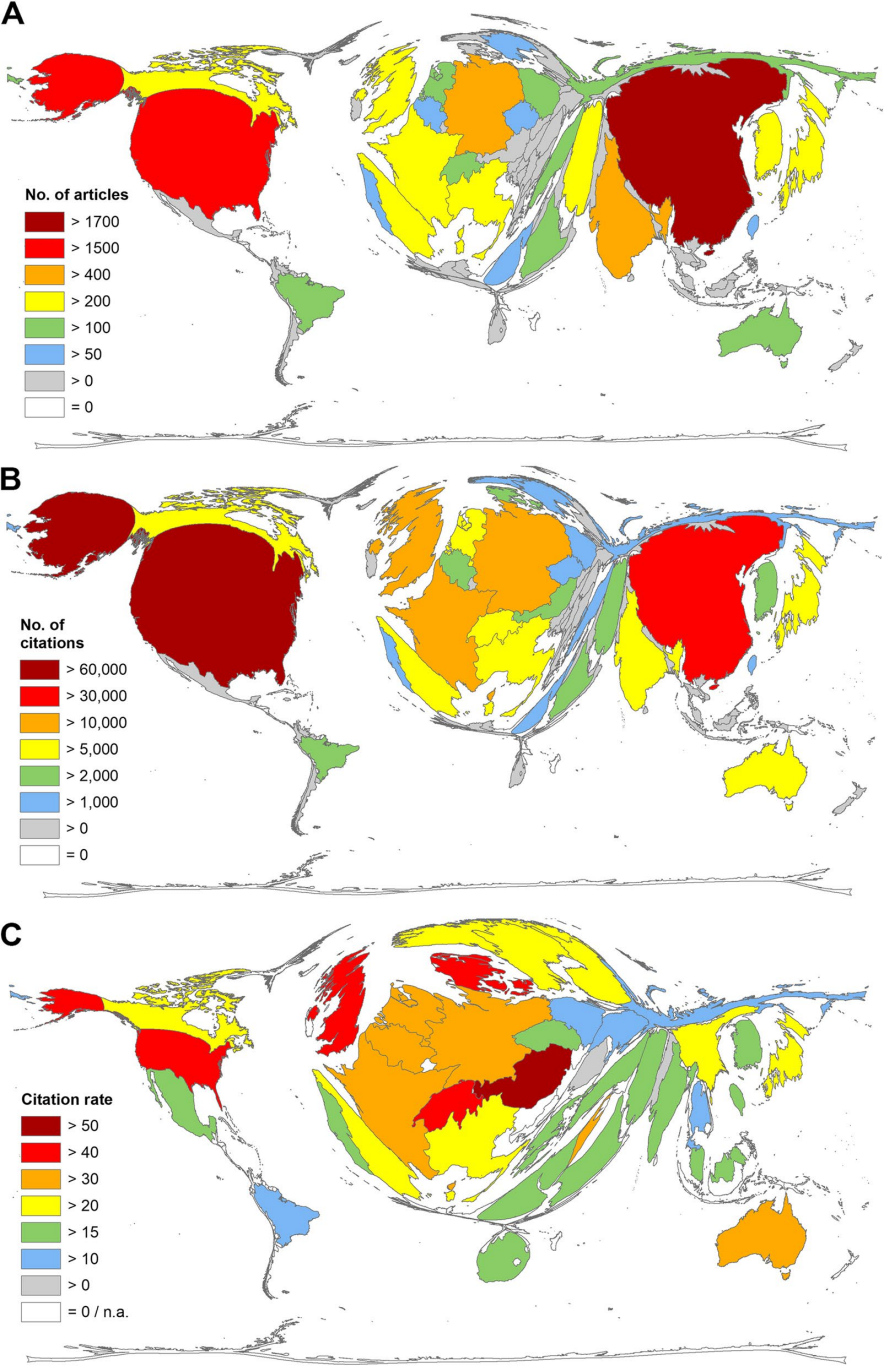
National publication output. **A** Article numbers. **B** Citation numbers. **C** Citation rate (threshold at least 30 articles on REE_eh_)

Of all retrieved articles, the vast majority, *n* = 6918 (99.67%), could be assigned to at least one country of origin. Of these, China and the USA are by far the most publishing countries at REE_eh_, with more than 1500 articles each (China *n* = 1730, USA *n* = 1558). Germany follows in third place by a wide margin with *n* = 478. Ranks 4 and 5 were occupied by India (*n* = 444) and Japan (*n* = 345). Of note is Iran’s ranking of 11th with *n* = 225 and Russia’s ranking of 15th with *n* = 153 articles on REE_eh_ (Fig. 4A).

The two major players also lead in the number of citations, but here the USA is in the lead and China follows at a greater distance (USA c = 63,286; China c = 35,379). This was followed by Germany (c = 17,766), the UK (c = 14,532), and France (c = 11,314) (Fig. 4B). The UK ranked only 8th in the number of publications (*n* = 341) but had the 4th highest citation rate of countries with at least 30 articles on REE_eh_. Austria ranked first in citation rate (cr = 58.25), followed by Denmark (cr = 46.80), and Switzerland (cr = 42.94). The UK (cr = 42.62) is followed by the USA in 5th place (cr = 40.62) (Fig. 4C).

**Fig. 5:**
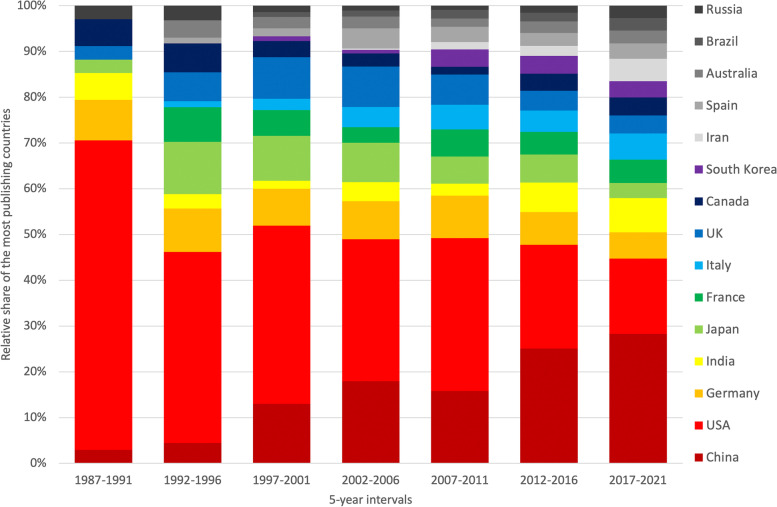
Relative share of the 15 most publishing countries in 5-year intervals from 1987 to 2021

The national share of publications has changed over time. The analysis of the contribution of most publishing countries shows the decreasing relative share of the USA and the simultaneously increasing relative share of China. In the first evaluation interval (1987-1991), only a few countries participated in REE_eh_ research, with the US being by far the most publishing country. In the last evaluation interval (2017-2021), the US-American share among the top 10 countries decreased to 16.47%, while China’s share increased to 28.27% (Fig. 5). The participation of India and Iran also increased steadily to 7.50 and 4.91%, respectively, in the last interval.

**Fig. 6:**
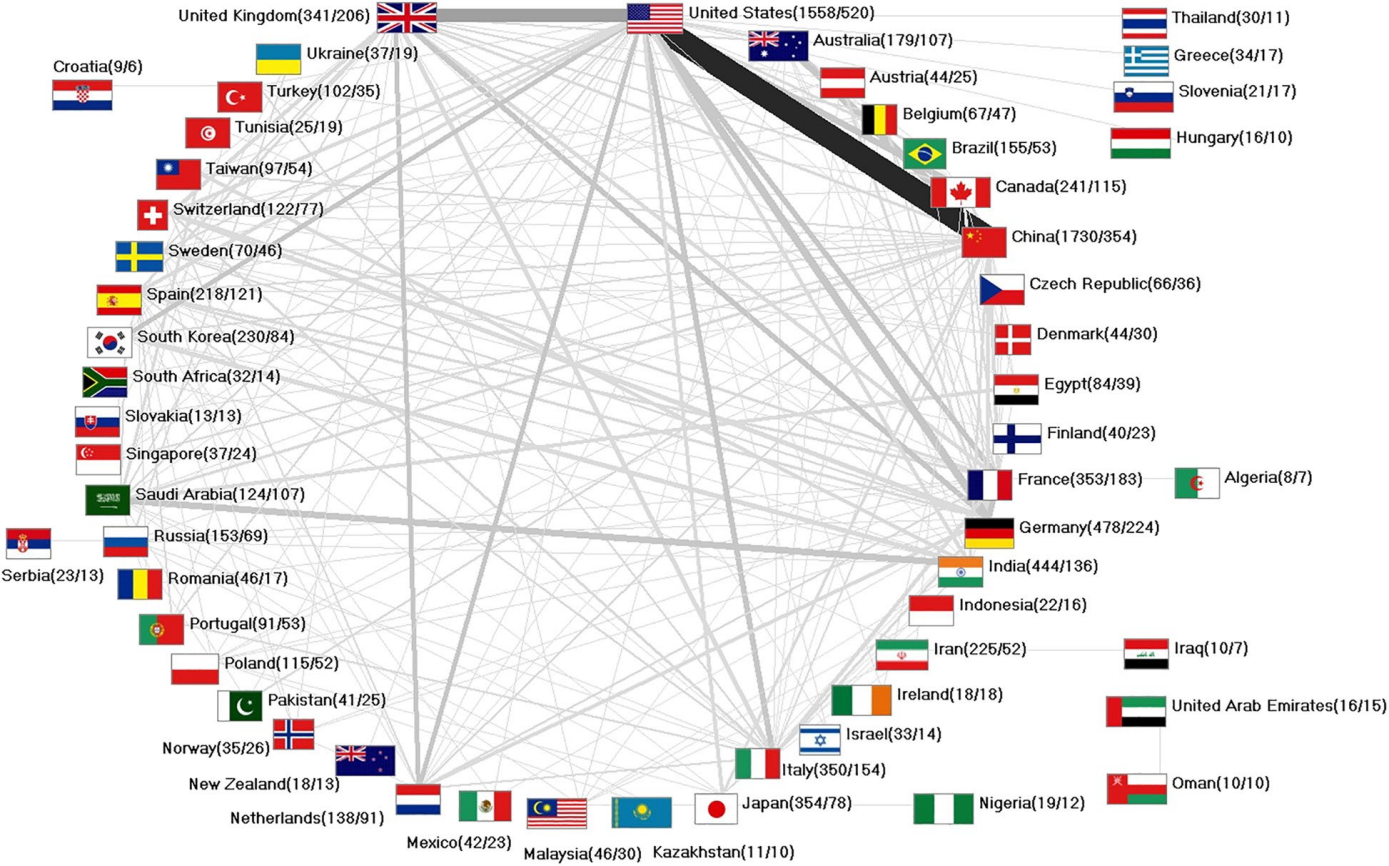
International research network, number in brackets (number of articles on REE_eh_ / number of collaboration articles on REE_eh_), display threshold = at least 5 collaboration articles

Over time, a broad international network has also developed for REE_eh_ research. The USA, as the core country, has generated the most collaborations. They collaborated primarily with China (*n* = 133) and the UK (*n* = 74). However, joint work with Germany (*n* = 46) and Canada (*n* = 39) is also notable. In addition, the partnerships between China and Australia (*n* = 41) and Saudi Arabian and Indian researchers (*n* = 36) are worth mentioning (Fig. 6).

### National foci

**Fig. 7:**
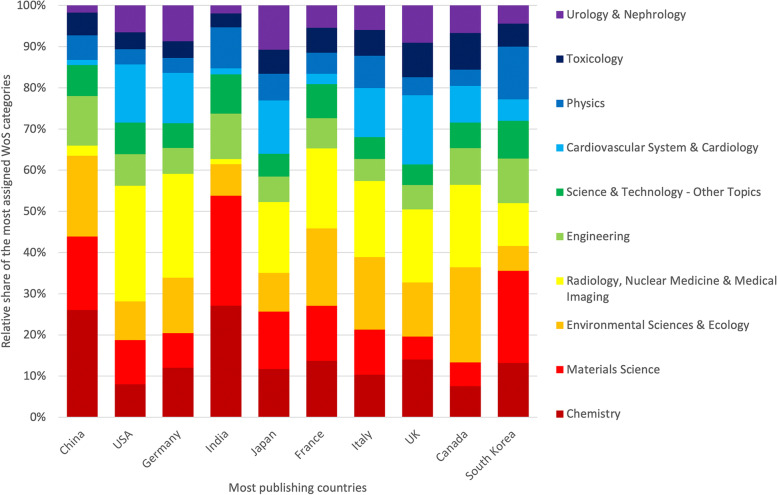
The relative share of the most assigned WoS categories of the most publishing countries

The most frequently addressed areas in this analysis were represented differently in the individual countries. In China and India, the most frequently recognized category overall was *Chemistry*, with 26.04 and 27.12%, respectively. In contrast, in the USA (28.12%), the most publishing European countries (Germany: 25.24%, Italy: 18.50%, France: 19.43%, the UK: 17.76%), and Japan (17.21%), the most frequently assigned area was *Radiology, Nuclear Medicine & Medical Imaging*. In India and China, this field landed in the last places of the top 10 research areas. *Environmental sciences & Ecology* was most represented in Canada (23.11%), followed by France (18.79%). However, this field also ranked second in Chinese REE_eh_ research (19.59%). In Western countries and Japan, *Cardiovascular System & Cardiology* was the second most covered research area, while in China, India, and France, this area is rarely covered and falls to the last place of the top 10. The WoS category *Materials Science* was assigned most frequently in South Korean articles on REE_eh_ and ranked second in China and India (Fig. 7).

### Inclusion of research infrastructural features

When national scientific characteristics are included, the order of the leading countries changes. When calculating R_GERD_ (ratio of the number of articles to gross R&D expenditures in billions of $), the ranking was topped by Iran, followed by Portugal, Pakistan, Ukraine, and Romania. When calculating R_RES_ (ratio of the number of articles to the number of researchers in 10,000 FTEs), Switzerland ranked first, followed by Romania, Italy, Portugal, and Iran (Table 3).

**Table 3:** Ranking of countries with at least 30 articles on REE_eh_ (threshold) of R_GERD_ (ratio of the number of articles and the Gross Expenditure on Research and Development (GERD) in bn $ ) and R_RES_ (ratio of the number of articles and the number of researchers in 10,000 Full Time Equivalents (FTE))

Country	GERD in bn $)	R_**GERD**_	Country	Researcher in 10,000 FTE	R_**RES**_
Iran	9.7362	23.11	Switzerland	4.6088	26.47
Portugal	4.4961	20.24	Romania	1.7518	26.26
Pakistan	2.2454	18.26	Italy	14.0378	24.93
Romania	2.6802	17.16	Portugal	4.4938	20.25
Ukraine	2.2614	16.36	Iran	11.8987	18.91
Egypt	7.2172	11.64	Czech Republic	3.9181	16.85
Italy	34.6576	10.10	Netherlands	8.3187	16.59
Spain	22.3193	9.77	Spain	13.3213	16.36
Poland	11.8447	9.71	Canada	15.8890	15.17
Greece	3.5385	9.61	Egypt	6.5301	12.86
Czech Republic	7.3024	9.04	Belgium	5.4010	12.41
Canada	29.6596	8.13	France	29.5754	11.94
India	55.1270	8.05	UK	28.9674	11.77
Australia	22.5552	7.94	Germany	41.9617	11.39
Netherlands	18.8032	7.34	USA	143.4415	10.86
UK	51.0291	6.68	South Africa	2.9515	10.84
Switzerland	19.1115	6.38	Finland	3.7047	10.80
Finland	7.1491	5.60	Norway	3.3632	10.41
France	66.0449	5.34	Poland	11.4585	10.04
South Africa	6.0256	5.31	China	174.0442	9.94
Mexico	8.1125	5.18	Greece	3.5000	9.71
Norway	6.9721	5.02	Denmark	4.5428	9.69
Brazil	33.0113	4.70	Sweden	7.3132	9.57
Turkey	21.7440	4.69	Singapore	3.8829	9.53
Denmark	9.6826	4.54	Austria	4.7521	9.26
Belgium	15.3555	4.36	Turkey	11.1893	9.12
China	420.8156	4.11	Ukraine	4.2164	8.78
Sweden	17.8373	3.92	South Korea	38.3100	6.00
Russia	42.3757	3.61	Pakistan	6.9769	5.88
Singapore	10.2552	3.61	Japan	67.6292	5.23
Germany	134.4298	3.56	Russia	41.0617	3.73
Austria	14.6550	3.00	Thailand	9.3457	3.21
USA	548.9840	2.84	Switzerland	4.6088	26.47
South Korea	90.3861	2.54	India	–	–
Thailand	12.0784	2.48	Australia	–	–
Japan	166.1837	2.13	Brazil	–	–
Israel	16.3525	2.02	Israel	–	–

### Inclusion of characteristics of global REE production

The inclusion of economically important market variables such as the national stock of electric vehicles, the number of wind turbines, and the production of permanent magnets led to another ranking of the publishing countries (Table 4).

**Table 4:** Ranking of countries with at least 30 articles on REE_eh_ (threshold) of R_EV_ (ratio of the number of articles and national stock of electric vehicles in 1000), R_PM_ (ratio of the number of articles and the export trade volume of permanent magnets in mill. $), and R_WPP_ (ratio of the number of articles and the number of wind power plants)

Country	Electric Vehicles (1000 no.)	R_**EV**_	Country	Trade Value Perm. Magnets (Mio. $)	R_**PM**_	Country	Wind power plants 2018 (no.)	R_**WPP**_
India	12.74	34.85	Pakistan	0.010522	3896.60	Iran	282	0.80
Brazil	4.94	31.35	Greece	0.106966	317.86	Czech Rep.	317	0.21
South Africa	1.40	22.87	Ukraine	0.145431	254.42	South Korea	1302	0.18
Greece	3.31	10.26	Turkey	0.410472	248.49	Japan	3661	0.10
Australia	26.65	6.72	Portugal	0.385775	235.89	Egypt	1190	0.07
Poland	18.88	6.09	Brazil	0.711452	217.86	Ukraine	533	0.07
Mexico	7.25	5.79	Russia	1.047843	146.01	Taiwan	1723	0.06
Italy	99.54	3.52	Spain	2.651529	82.22	Thailand	778	0.04
Spain	88.01	2.48	Israel	0.614	53.75	Italy	9958	0.04
Portugal	49.70	1.83	Australia	3.915023	45.72	Pakistan	1189	0.03
South Korea	136.55	1.68	Canada	5.640438	42.73	Australia	5362	0.03
Switzerland	86.47	1.41	France	8.756324	40.31	Netherlands	4471	0.03
Japan	293.08	1.21	South Africa	1.069684	29.92	France	15,309	0.02
Canada	209.08	1.15	Norway	1.183535	29.57	Norway	1675	0.02
USA	1778.09	0.88	India	18.520671	23.97	Belgium	3360	0.02
France	416.21	0.85	Italy	16.168699	21.65	Poland	5864	0.02
Germany	633.42	0.75	Poland	5.814926	19.78	Finland	2041	0.02
UK	455.03	0.75	Belgium	3.510913	19.08	Canada	12,816	0.02
Finland	55.32	0.72	USA	94.193148	16.54	Portugal	5380	0.02
Denmark	61.61	0.71	Sweden	4.786267	14.63	UK	20,970	0.02
Belgium	104.40	0.64	UK	27.345533	12.47	USA	96,665	0.02
Netherlands	291.13	0.47	South Korea	22.338295	10.30	South Africa	2085	0.02
Sweden	178.71	0.39	Czech Rep.	7.766101	8.50	Romania	3029	0.02
China	4508.67	0.38	Austria	7.146708	6.16	Austria	3045	0.01
Norway	484.67	0.07	Finland	11.854787	3.37	Turkey	7369	0.01
Austria	n.s.	–	Singapore	11.301057	3.27	India	35,129	0.01
Czech Rep.	n.s.	–	Denmark	16.844438	2.61	Greece	2844	0.01
Egypt	n.s.	–	Germany	217.516163	2.20	Brazil	14,707	0.01
Iran	n.s.	–	Switzerland	64.41377	1.89	Sweden	7407	0.01
Israel	n.s.	–	Netherlands	72.895252	1.89	Spain	23,494	0.01
Malaysia	n.s.		Japan	384.483454	0.92	Mexico	4935	0.01
Pakistan	n.s.	–	China	2037.45097	0.85	China	211,392	0.008
Romania	n.s.	–	Malaysia	68.956219	0.67	Germany	59,311	0.008
Russia	n.s.	–	Thailand	59.110702	0.51	Denmark	5758	0.008
Saudi Arabia	n.s.	–	Iran	n.s.	–	Russia	n.s.	–
Singapore	n.s.	–	Saudi Arabia	n.s.	–	Saudi Arabia	n.s.	–
Taiwan	n.s.	–	Taiwan	n.s.	–	Switzerland	n.s.	–
Thailand	n.s.	–	Egypt	n.s.	–	Malaysia	n.s.	–
Turkey	n.s.	–	Romania	n.s.	–	Singapore	n.s.	–
Ukraine	n.s.	–	Mexico	n.s.	–	Israel	n.s.	–

**Fig. 8:**
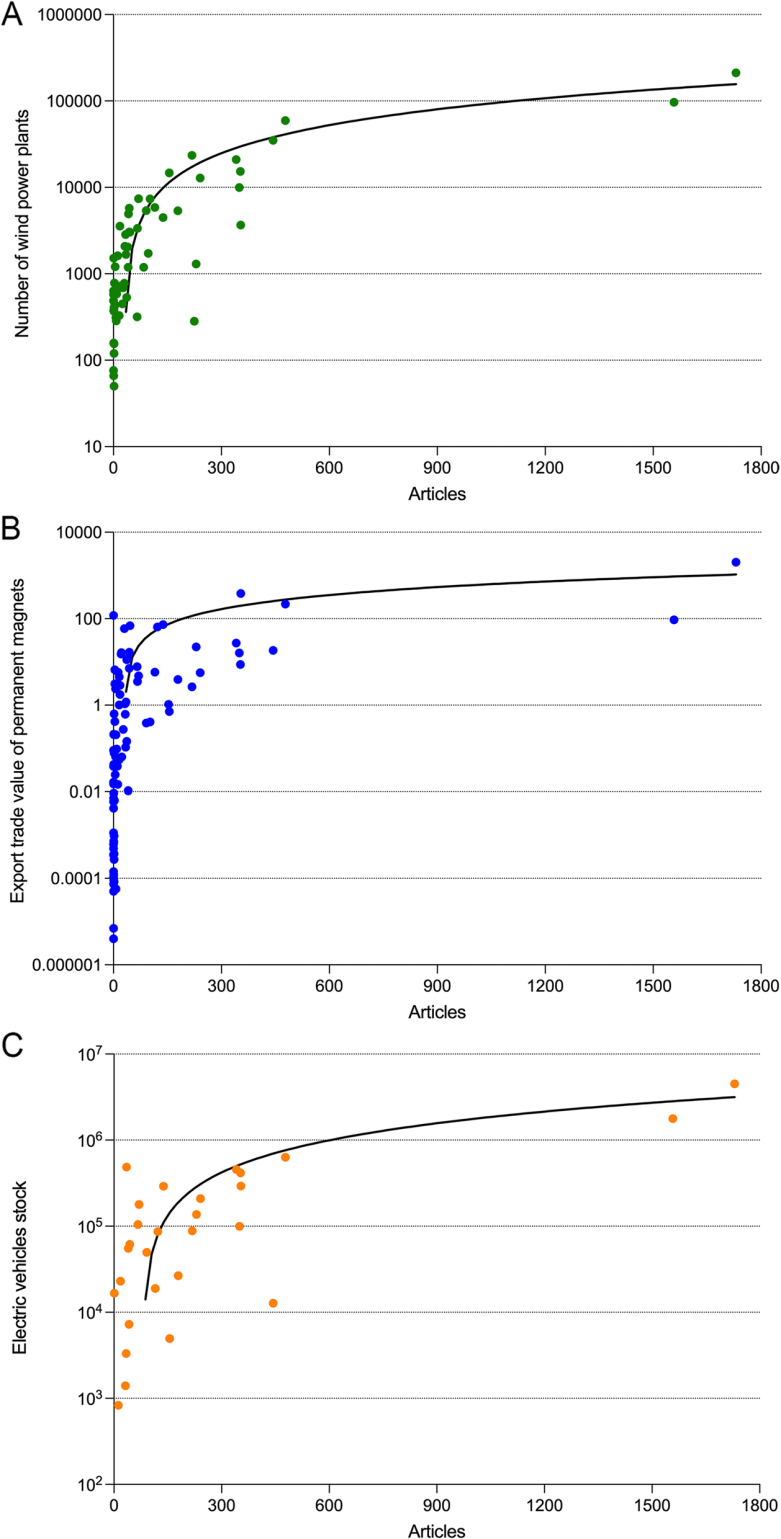
Linear regression (Spearman) of the number of articles on REE_eh_ and market driver parameters **A**) Number of wind power plants (61 countries included), **B** Export trade value of permanent magnets in mill. $ (92 countries included), **C** Electric vehicles stock (28 countries included)

To determine the correlation between the number of articles on REE_eh_ and market values, the correlation and the linear regression with the residual (Spearman) (Fig. 8) were calculated. All correlations were highly significant (articles and wind turbines: *r* = 0.80, *p* < 0.0001; articles and export trade value of permanent magnets: *r* = 0.78, *p* < 0.0001; articles and electric vehicles: *r* = 0.65, *p* = 0.0002).

### Most publishing institutions

The institutions that mainly work on REE_eh_ are summarized in Table 5.

**Table 5:** The 10 top publishing institutions measured by the number of articles on REE_eh_

Institution	Country	Articles	Citations	Citation rate
Chinese Academy of Science	China	276	7913	28.67
Harvard University	USA	85	5139	60.46
Russian Academy of Science	Russia	81	732	9.04
Peking University	China	69	1521	22.04
University of London	UK	67	4548	67.88
Sun Yat Sen University	China	62	1370	22.10
Mayo Clinic	USA	62	3788	61.10
King Saud University	Saudi Arabia	59	869	14.73
Shanghai Jiao Tong University	China	56	1039	18.55
Imperial College London	UK	52	3799	73.06

## Discussion

### Aspects of research development and focus

For the publication output on REE_eh_, a total of 6941 original articles indexed in WoS could be identified. They can be grouped according to the occurrence of keywords in some main topic areas, which are headed by the term “toxicity”. The first article on REE_eh_ published in 1950 by Cochran et al. addressed the acute toxicity of some REEs and found that the general toxicity observed in rats was consistent with the similarity of the elements [20]. This study has been cited repeatedly over time, 82 times to date, which is a comparably high number for postwar publications. In the 1960s, the research-grade REEs became available in sufficient amounts and at moderate prices [1] leading to a small over-proportional publication output compared to all SCIE-articles. In 1961, a study on the toxicological effects of gadolinium and samarium chlorides, which contained some evidence of adverse health effects, was also highly cited, with 69 citations [21].

The next study with a substantial number of citations (c = 420) was published in 1990. It examined the influence of thermodynamics on the toxicity of gadolinium complexes to increase their inherent safety [22]. This publication marked the beginning of an initially moderate and, from 2005, steep increase in work on REE_eh_. In 2008 and 2015, both years with major citation peaks, particularly high-cited articles were published, including the top-cited studies of Xia et al. on cerium oxide toxicity [19] and McDonald et al. on gadolinium deposition after MRI [23]. This also points to the identified thematic focus around the term “nanoparticle” in relation to oxidative stress and cytotoxicity, particularly of cerium oxide, and the term “gadolinium” in relation to its risks associated with use as a contrast agent in MRI.

In the 1950s, the nuclear arms race gave impetus to REE production and research. The opening of mines in Russia, South Africa, and California (USA) in the early 1950s was caused by the rising value of REEs. The contrast-enhancing effects of gadolinium were discovered in the 1980s [24], which later spurred research on its adverse health effects and triggered the steep increase in publications since 1990, as evidenced by the appearance of, for example, the article by Cacheris et al. [22]. The disproportionate trend from 1990 compared to SCIE articles establishes this relationship. The adverse health effects of gadolinium, which can cause nephrogenic fibrosis, identified by Grobner [25] 2006, led to research interest in this area and an increased volume of annual publications subsequently. In 2008, a year with a clearly visible citation spike, CeNPs were in the focus of two of the most cited articles in this analysis [19, 26]. Both analyzed the effect of CeNPs on the cell, noting or focusing on a protective effect against oxidative stress. In 2006 and 2011, the articles by Schubert et al. [27] and Celardo et al. [28], which are likewise among the most-cited articles, also addressed the neuroprotective and pharmacological potential of CeNPs.

After this year of high interest in the scientific community in 2008, the number of annual citations declined from 2009 to 2012, representing an apparent interim low point analogous to the declining depletion of REEs. At the same time, the number of publications continued to increase at a high level. In 2008, the share of Chinese articles was rather low compared to the years before or the steady increase after. This rise led China to overtake the USA in terms of the number of publications in 2013. Thus, it is to assume that the trend during this period is not due to an actual drop in citations, but more to the extremely high response in 2008, which was mainly due to the high proportion of US-American and German articles. Accordingly, China’s share of articles assigned to the subject area “Radiology, Nuclear Medicine & Medical Imaging” is small, while US-American and German research is primarily focused on this area. The trend, except for 2008, seems to be coherent. Since 2012, the growth of publications on REE_eh_ has been extremely high, leading to a citation peak in 2015 with two high-profile articles, Kanda et al. [29] and McDonald et al. [23]. Both articles deal with the deposition or accumulation of gadolinium in the brain.

The subsequent decline in citation numbers is due to the methodological phenomenon of the so-called cited half-life (CHL). This concept describes the estimated time it takes for a publication to reach 50% of its citations. For biomedical research, this period is assumed to be 7-8 years corresponding to the CHL [30]. The citation rate still decreases as the number of Chinese publications increases. This is evidenced by the earlier onset of the falling values compared to the CHL-induced drop. This will certainly change in the future, as Chinese researchers are now aware of the importance of impact factors and other bibliometric key figures. They are obliged by governmental policies to produce not only quantity, but also high-quality studies that are listed in WoS [31, 32].

### Global research aspects

The high share held by Chinese and US-American REE_eh_ research is not surprising, as both countries have been competing for key positions in research performance in recent years [31]. The main citation figures are currently still concentrated in the USA. Although, this may change in the future. Until the mid-1980s, the main source of REEs was in California, USA. But this mine was closed in 2002 due to stiff competition from emerging Chinese mining and the environmental problems associated with REE mining [1]. Russian production was also scaled back during this period. The USA and Russia later started the REE mining again [33]. Besides these two countries, some European countries (Germany, France, Italy, UK), India, and Japan dominate the global research landscape. It can be seen that the high-income countries focus mainly on radiological topics. “Nephrology” is also more prevalent in the REE_eh_ research in these countries. They focus on the health effects of gadolinium as a contrast agent.

In comparison, China and India focus more on chemical and material topics. In China and Canada, environmental issues also receive high attention. For this, China, with the most production facilities, is clearly in the best position to study the hazardous effects of unregulated production. The results of these studies led to a rethink by Chinese authorities and the introduction of regulations and restrictions. So, China has shut down some small-scale mines and thinks about gentle mining procedures and the prevention of illegal mining through the Chinese consolidation strategy [34] to reduce adverse impacts on human health, ecotoxicity, and eutrophication and acidification of soils [35]. To what extent this will lead to an improvement in the environmental impact of REE mining remains to be seen.

Regarding the ranking in terms of the citation rate, Austria is leading. With 1261 citations alone, the article by Thomas Grobner on the health risk of gadolinium [25] was responsible for this, especially since Austria having 44 articles on REE_eh_ was only just above the analysis threshold of 30 articles. Grobner is a physician and specialist in internal medicine but not a high-profile scientist, having achieved only an h-index of 6 so far. Nevertheless, he set a milestone with his scientific observations in REE_eh_ research.

Denmark (*n* = 44), which ranked second in terms of citation rate, also achieved this rank with research on the health effects of gadolinium, e.g., during pregnancy and lactation [36]. Some of these studies were carried out in collaboration with Switzerland [37], which followed in third place in terms of citation rate. With partial participation of Danish scientists, Swiss research has successfully analyzed the use of yttrium additives for radioimmunotherapy of follicular lymphoma. This is the most common form of NHL, which has been shown to cause hematologic toxicities [38]. Switzerland also topped the rankings when the number of researchers per country was included.

Regarding international collaborations, it is noteworthy that Saudi Arabia has been India’s primary partner in REE_eh_ research. One focus of their joint work is the function of some REEs, e.g., for photo-degeneration or the -degradation of environmental pollutants [39, 40]. Apart from these collaborations, most of the cooperation took place between the two major players, China and the USA. Despite their different research foci, they share common interests in the field of environmental science, primarily because of the opportunity to study impacts directly at mining sites, the most located in China (Table 6).

**Table 6:** Global REE mine production 2021* (Metric tons Rare Earth Oxide Equivalents), * estimated, ** undocumented production not included [33]

Country	%
China **	60.63
USA	15.52
Myanmar	9.38
Australia	7.94
Thailand	2.89
Madagascar	1.15
India	1.05
Russia	0.97
Brazil	0.18
Vietnam	0.14
Burundi	0.04
Rest of world	0.11

Among REE mining countries, also India is in the top 5 of the publication ranking. It is also leading when the national economic interest concerning the stock of electric vehicles is included in the comparison. India has a long tradition of REE mining, as the government established *India Limited* (IREL), formerly *Indian Rare Earth Limited* [41], back in 1950.

Looking at other countries that mine REE and are ranked in the top 15 of the publication ranking, Brazil, has a long tradition of mining, as does Russia. The former USSR had long mined REEs with its mines in Kyrgyzstan [33]. Scientific collaboration between Kyrgyz, Ukrainian, and Russian scientists is due to their shared history and the close ties that still exist. After closing its mines in 2003, Russia started exploiting REEs again in 2008 [33], which was accompanied by increasing publication numbers. The strong scientific partnership between Russia and Ukraine will certainly be affected during and after Russia’s war of aggression against Ukraine.

Other mining countries, Myanmar, Madagascar, Vietnam, and Burundi play rather minor roles in REE_eh_ research. In Madagascar and Burundi, mining only started in 2017 and 2018, respectively [18], so their low contribution to REE_eh_ research can be explained.

In addition to the influence of the countries’ mining status, the strong relationship between publication output and proxy values for market drivers (electric vehicle inventory, trade value of permanent magnets, and number of wind turbines) was demonstrated by significantly high correlation values.

In addition to these ratings, Iran tops the ranking when national research expenditures (GERD) are included in the evaluation of countries’ research performance in REE_eh_. It also ranks at the top when including interest in wind energy as measured by the number of national wind turbines. The Iranian government announced in early 2020 that it intends to industrialize the production of REEs [42] – an effort initiated in 2017 at the 9th Symposium of the *Iranian Society of Economic Geology* [43]. Notably, studies on the suitability of potential mining sites increased accordingly in Iran [44], especially after the extraction of REEs as a byproduct of uranium mining was considered [45]. This decision was accompanied by increase in research on REE_eh_ in Iran.

Thus, the major publishing countries in absolute terms, the USA and China, fell behind when economic characteristics or the number of researchers per country were taken into account. As nations with the highest science funding and the highest number of researchers worldwide, this drop is not surprising when the ratio to publication output is calculated.

Instead, Portugal ranked second in the GERD rankings after Iran. Environmental research is at the heart of Portuguese studies on REE_eh_. For example, studies have been conducted in the Tagus Estuary Nature Reserve, one of the most important wetlands in Europe, or in other water bodies to determine REE contamination as an indicator of anthropogenic activities. Another Portuguese research focus was the accumulation of REEs in the environment. It was investigated where the REEs come from, whether from near inactive chemical complexes, effluents from wastewater treatment, or lithogenic sources, as in a coastal lagoon near Aveiro. The University of Aveiro was the institution where most Portuguese research on REE_eh_ has been conducted [46–48].

Another country worth mentioning in terms of REE_eh_ publication is Pakistan. It ranks third in regard to the inclusion of GERD and first in the inclusion of the trade value of permanent magnets. Even after REE deposits were discovered in Pakistan, the government did not initially promote mining. The reason given was the lack of financial resources and technical expertise for exploitation, although the reserves were highly estimated. A shift in thinking in this regard began in 2010 and coincided with the start of REE research in Pakistan. But it was not until 2021 that Pakistani publications reached double digits for the first time, at a time when the government finally decided to exploit its REE reserves [49].

## Conclusions

Taking all these historical and regional aspects into account, it is clear that the research efforts on REE_eh_ depend on economic factors on the one hand and interests and accessibility to data from mining sites on the other hand. The exploitation of REEs is still in the hands of a few countries, first and foremost China. Therefore, obtaining data is difficult for independent and open-ended research is difficult, which is necessary for research on the environmental and health risks of REE use and production. In addition, an independent and secure supply of REE must be maintained. Substitutes or reduction possibilities must be sought for the REEs applied, like those currently available are generally even less effective. New separation technologies for energy-saving processes must be sought. There is also a need for research in the area of recycling REE-containing equipment, as only a very small proportion is reused to date. All of these mandatory tasks represent an enormous challenge for future research that can only be met with networked expertise and resources on a global scale.

## Method

### Methodological platform, data source, and data base generation

This analysis is based on the methodological and technical principles developed by the *New Quality and Quantity Indices in Science* (NewQIS) platform, which is an established methodology for assessing scientific performance under a variety of basic [50] and advanced parameters [51]. It combines a representation of the global scientific landscape with sophisticated visualization techniques such as density equalizing map projections (DEMP) by following a method by Gastner and Newman [52].

The metadata underlying the bibliometric analyses was taken from the Web of Science (WoS) Core Collection, one of the most representative and qualitative sources of bibliometric research.

The data collection took place on 04-08-2022 and included all years from 1900 onwards.

The search strategy included, first, a title search with the various denominations for REE and all elements declared as REE. Only the radioactive promethium was excluded from the search because it does not form stable isotopes and cannot be extracted from geological material because it occurs only in “infinitesimal” concentrations [1, 53].

1. Title: “rare earth element*” OR “rare earth metal*” OR “tech metal*” OR “high-tech metal*” OR “cerium” OR “dysprosium” OR “erbium” OR “europium” OR “gadolinium” OR “holmium” OR “lanthanum” OR “lutetium” OR “neodymium” OR “praseodymium” OR “samarium” OR “terbium” OR “thulium” OR “ytterbium” OR “yttrium” OR “scandium” OR “lanthanid*” OR “lanthanoid*”.

Second, a combination of relevant terms describing human and environmental health effects of REEs was searched as a topic search in the title, abstract, and keywords. These terms were combined with the title search.

2) Topic: “Risk*” OR “contamination*” OR “hazard*” OR “*toxicolog*” OR “*toxicit*” OR “adverse effect*” OR “negative effect*” OR “negative health effect*” OR “*toxic effect*” OR “ecological” OR “pollution*” OR “contaminant*” OR “pollutant*” OR “bioaccumulat*” OR “bio-accumulat*” OR “bioavailab*” OR “bio-availab*” OR “threat*” OR “ecology” OR “environmental impact*” OR “oxidative stress” OR “fibrosis” OR “fibrotic” OR “male sterility” OR “pneumoconiosis” OR “neurological disorder*” OR “anti-testicular effect*”

Entries were filtered for original articles and then manually checked for representativeness. Subsequently, the term “price” had to be excluded from the search because it returned not thematically related entries. All metadata were transferred in a structured manner to an MS-Access database, which served as the basis for all analyses performed. Afterward, some data had to be manually standardized, such as the variations in the designation of affiliation, to unify them and thus make them suitable for further analysis.

### Performed analyses and visualization techniques

The analyses included absolute parameters such as annual publication parameters, the number of publications, the number of citations, the citation rate per country, and research institutions to identify the major players in REE_eh_ research worldwide. Relative parameters such as the characteristics of the scientific infrastructure and the main drivers of rare earth exploration were additionally used for an enhanced assessment. For this purpose, the number of researchers and the gross expenditure on research and development (GERD) [54] were used on the one hand. On the other hand, the number of wind turbines in 2018 [55], the national stock of electric vehicles [56], and the trade value of permanent magnets [57] were included as approximate values for relevant market drivers.

The main research areas were identified and analyzed for clustering using the VOSviewer application [58] and visualized to create density maps of the occurring keywords. Additionally, the assigned WoS categories directing to the research areas. They are assigned to each listed journal by WoS. Every article adopts automatically the category assigned to the journal in which it is published. As there are multidisciplinary journals there are more subject categories than articles in number.

To visualize the geographic results, DEMPs were applied to the national publication patterns. Using this algorithm, the world map is distorted according to the particular evaluation parameter by increasing the size of countries with high values and decreasing the size of countries with respective low values.

#### Journals

Statistically, the relationship between national characteristics and the number of publications was calculated using linear regression and correlation analysis (Spearman).

### Methodological limitations and strength

The results presented have to be considered in context to the limitations and strength of the methods applied. WoS has been recommended for bibliometric analyses in numerous studies and has proven its worth by not only providing citation information but also ensuring database quality through strict listing requirements [59, 60]. Hence, it has advantages over the other databases that also provide citation counts, namely Scopus and Google Scholar. Scopus lists only articles since the 1990s, making it useless for the purpose of our study. Google Scholar lists all publications without quality requirements, while WoS requires an impact factor of the journal and proper peer review for the articles. Without these quality criteria, a valid scientific analysis is not possible because there is too much untested data. Another point to consider is the English bias of WoS, which is certainly responsible for the large proportion of English articles [61, 62].

The search strategy also has some limitations. It must be taken into account that, in principle, not all existing publications can be found and integrated into the analyses. However, the strategy can be optimized to find the majority of related entries and the minimum of unrelated entries. To ensure the representativeness of the database generated in this way, the metadata found were additionally checked manually and on a random basis.

## Data Availability

The bibliometric data is owned by and has been obtained from the Web of Science database. Therefore, authors are not allowed to share the data publicly or privately. Any researcher with access to the Web of Science database can obtain the data using the methods described in the paper.

## Abbreviations

CeNP: Cerium Oxide Nanoparticle
CHL: Cited Half-Life
DEMP: Density Equalizing Map Projections
EC: Emerging Contaminant
FTE: Full Time Equivalent
GERD: Gross Expenditures for Research and Development
IREL: India (Rare Earth) Limited
IUPAC: International Union of Pure and Applied Chemistry
MRI: Magnetic Resonance Imaging
NewQIS: New Quality and Quantity Indices in Science
NHL: Non-Hodgkin's Lymphoma
REE: Rare Earth Elements
REE_eh_: Rare Earth Element Research in context of environment and health
SCIE: Science Citation Index Expanded
WoS: Web of Science
